# Role of Presenilin-1 in Aggressive Human Melanoma

**DOI:** 10.3390/ijms23094904

**Published:** 2022-04-28

**Authors:** Julia Sidor, Megan Gillette, Lindsay Ann Dezi, Gustavo Untiveros, Luigi Strizzi

**Affiliations:** 1 College of Osteopathic Medicine, Midwestern University, Downers Grove, IL 60515, USA; jsidor12@midwestern.edu (J.S.); mgillette53@midwestern.edu (M.G.); 2 Department of Pathology, College of Graduate Studies, Midwestern University, Downers Grove, IL 60515, USA; dezilindsay@gmail.com (L.A.D.); guntiv@midwestern.edu (G.U.)

**Keywords:** presenilin-1, melanoma, biomarker, Wnt signaling

## Abstract

Presenilin-1 (PS-1), a component of the gamma (γ)-secretase catalytic complex, has been implicated in Alzheimer’s disease (AD) and in tumorigenesis. Interestingly, AD risk is inversely related to melanoma, suggesting that AD-related factors, such as PS-1, may affect melanomagenesis. PS-1 has been shown to reduce Wnt activity by promoting degradation of beta-catenin (β-catenin), an important Wnt signaling partner. Since Wnt is known to enhance progression of different cancers, including melanoma, we hypothesized that PS-1 could affect Wnt-associated melanoma aggressiveness. Western blot results showed that aggressive melanoma cells expressed significantly lower levels of both PS-1 and phosphorylated-β-catenin (P-β-catenin) than nonaggressive melanoma cells. Immunohistochemistry of human melanoma samples showed significantly reduced staining for PS-1 in advanced stage melanoma compared with early stage melanoma. Furthermore, γ-secretase inhibitor (GSI) treatment of aggressive melanoma cells was followed by significant increases in PS-1 and P-β-catenin levels, suggesting impaired Wnt signaling activity as PS-1 expression increased. Finally, a significant reduction in cell migration was associated with the higher levels of PS-1 and P-β-catenin in the GSI-treated aggressive melanoma cells. We demonstrate for the first time that PS-1 levels can be used to assess melanoma aggressiveness and suggest that by enhancing PS-1 expression, Wnt-dependent melanoma progression may be reduced

## 1. Introduction

Melanoma develops from the malignant transformation of melanocytes, the neural crest-derived, pigment-producing cells of the skin [1,2]. Despite making up only 1% of all skin cancers, it accounts for almost the entirety of skin cancer-related deaths, with a dismal 5-year survival rate of only 27% for metastatic disease [1,2,3]. However, with early detection and excision of in situ lesions, the 5-year survival rate of melanoma significantly improves to 99% [1].

The progression of melanoma from in situ, radial growth phase (RGP) to locally infiltrating vertical growth phase (VGP) is multifactorial and influenced by aspects that include but are not limited to: environmental factors such as ultraviolet radiation; genetic mutations of tumor suppressor genes such as *TP53*; mutations that lead to constitutively active signaling of molecules such as those of the mitogen-activated protein kinase (MEK) pathway; and epigenetic disturbances of gene transcription, such as DNA methylation [4,5,6]. Undoubtedly, this complexity creates a challenge in managing melanoma, as targeting one pathway invariably leads to activation or selection of cells with alternative signaling that continue to drive melanoma progression [5,7,8].

There has been continued interest in exploring how neurodegenerative diseases and associated cellular and microenvironmental factors can affect tumorigenesis. For instance, patients diagnosed with melanoma showed decreased risk for Alzheimer’s disease (AD), encouraging research focused on identifying factors involved in pathogenesis of AD that could also affect melanoma growth [9,10]. Although the exact cause of AD is still unknown, current theory suggests that dysfunctional processing of amyloid-β precursor protein (APP) results in the formation of β-amyloid peptide (Aβ) that accumulates and creates extracellular neuritic plaques, which are neurotoxic and believed to act as a catalyst for development of AD [11,12,13]. AD is classified into two main categories: late-onset, or sporadic, type (SAD) and early-onset, or familial, type (FAD) [14,15]. Both types share the same pathological features and pathogenic events that lead to dysfunctional APP processing [11,12]. While no specific gene has been linked to SAD, there are distinct genetic variants that cause FAD, such as mutation of *PSEN-1* [11,14]. *PSEN-1* encodes presenilin-1 (PS-1), a ubiquitously expressed multipass transmembrane protein involved in APP processing and other molecular functions [11,14].

PS-1 is commonly known for its role, in conjunction with presenilin-2 (PS-2), as the catalytic core of the gamma (γ)-secretase complex, an intramembrane aspartyl protease complex, the substrates of which include the amyloid-β precursor protein (APP) [12]. Mutations, whether they result in decreased levels of functional PS-1 or increased expression of dysfunctional PS-1, can lead to aberrant processing of APP that is involved in the pathogenesis of FAD [12]. Initially, γ-secretase inhibitors (GSI), such as DAPT (N-[N-(3,5-Difluorophenacetyl)-L-alanyl]-S-phenylglycine t-butyl ester ), were used to target Aβ production for treatment of AD, but results were unsuccessful, in part because of rebound increases in PS-1 expression with continued aberrant processing of APP and possibly additional γ-secretase-independent PS-1 functions such as intracellular calcium signaling, autophagy degradation, and turnover of the important Wnt signaling cofactor β-catenin [11,13]. In fact, PS-1 negatively regulates Wnt signaling by promoting phosphorylation and subsequent degradation of β-catenin [11,16,17]. In this regard, PS-1 has been shown to bind to β-catenin and then recruit kinases, such as glycogen synthase kinase-3, into the PS-1/β-catenin complex, causing phosphorylation of β-catenin (P-β-catenin) at Ser33/37/Thr41 [18,19]. PS-1 can also induce phosphorylation of β-catenin at Ser45 [20]. Interestingly, Liu et al. demonstrated that phosphorylation of β-catenin at Ser45 then leads to phosphorylation at Ser33/37/Thr 41 [21]. Thus, detection of P-β-catenin Ser33/37/Thr41 implies that β-catenin is also phosphorylated at Ser45. Regardless of the different phosphorylated sites, it is important to note that P-β-catenin Ser33/37/The41 is the form that is ultimately recognized for ubiquitination and proteasomal degradation [22,23]. It is not clear, however, how quickly ubiquitination and proteasomal degradation occur, especially in cancer cells, before significant reductions in total cellular β-catenin levels can be detected.

Wnt/β-catenin signaling is a highly conserved pathway that plays a critical role during development by controlling activities such as cell fate, organogenesis, and regulation of cell migration and polarity [24]. The Wnt/β-catenin signaling pathway is activated by Wnt ligands, which are secreted glycoproteins that bind to the Frizzled receptor. This triggers the accumulation and translocation of β-catenin to the nucleus, where it acts as a transcriptional coactivator of genes involved in cellular activities including differentiation, proliferation, and survival [24,25]. Different studies have reported increased Wnt/β-catenin signaling during melanoma progression [26,27]. Furthermore, it was shown that patient melanoma tissue samples with increased Wnt/β-catenin signaling were associated with reduced overall survival [28]. Thus, inhibiting Wnt/β-catenin signaling in melanoma can lead to therapeutic effects. However, targeting Wnt/β-catenin signaling in different cancers has been associated with unacceptable safety profiles as a result of off-target effects due to the ubiquitous expression of Wnt/β-catenin [29,30].

As mentioned above, Wnt function is dependent on β-catenin availability, and PS-1 can regulate Wnt activity by controlling β-catenin levels. Several studies have reported that decreased expression of PS-1 is associated with worse outcomes in certain cancers, such as breast, skin, and glioblastoma [17,31,32]. In skin, PS-1 deficiency led to an increase in β-catenin expression, which caused epidermal hyperplasia and tumor formation [31]. Similarly in glioblastoma, PS-1 showed an antiproliferative effect due to its ability to enhance β-catenin degradation by increasing P-β-catenin levels, which leads to repression of Wnt signaling [17]. It is reasonable to predict, therefore, that PS-1 may play a role as a potential tumor suppressor by regulating β-catenin availability for nuclear translocation and induction of Wnt signaling in cancer cells.

Here, we investigated the role of PS-1 in melanoma. We showed that increased melanoma aggressiveness was associated with reduced PS-1 expression. Moreover, we demonstrated that increasing PS-1 expression in melanoma cells led to reduced Wnt/β-catenin signaling and migratory potential. These results indicated a novel role for PS-1 as a biomarker for melanoma aggressiveness and repressor of melanoma progression.

## 2. Results

### 2.1. PS-1 Expression Was Lower in Aggressive Melanoma Cells with Active Wnt Signaling

**Figure 1:**
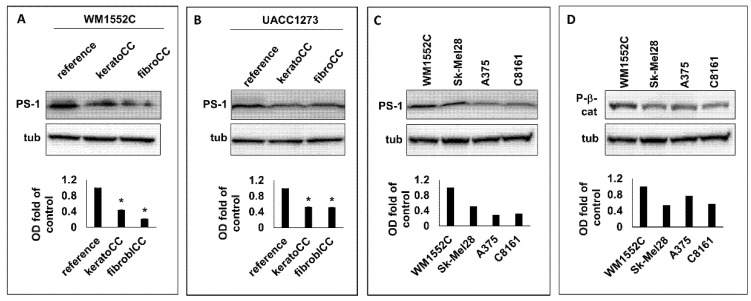
Western blot analysis of PS-1 and P-β-catenin in nonaggressive and aggressive melanoma cells. Western blot results with corresponding densitometric analysis of WB bands showed decreased PS-1 protein expression compared with control cells (reference) in two separate nonaggressive melanoma cell lines, WM1552C (**A**) and UACC1273 (**B**), after exposure to normal skin keratinocytes (keratoCC) and normal dermal fibroblasts (fibroCC) (* *p* < 0.05). (**C**) Western blot results with corresponding densitometric analysis of WB bands of cell lysates from aggressive melanoma cells (Sk-Mel28, A375, and C8161) showed lower PS-1 levels than results from nonaggressive melanoma cells (WM1552C). (**D**) Western blot results with corresponding densitometric analysis of WB bands also showed increased Wnt signaling, as demonstrated by the decreased levels of P-β-catenin (P-β-cat), in the aggressive versus nonaggressive melanoma cells analyzed.

We previously showed that nonaggressive melanoma cells exposed to normal skin cells acquired phenotypic and molecular characteristics of increased aggressiveness, which included enhanced cell migration and increased Wnt signaling [33]. Here, WB analysis was performed to ascertain whether PS-1, a known regulator of Wnt function, was implicated in Wnt signaling observed in nonaggressive melanoma cells exposed to normal skin cells. Our WB results (Figure 1A,B) showed a significant decrease (*p* < 0.05) in PS-1 expression in the nonaggressive melanoma cell lines WM1552C and UACC1273 (Supplementary Table S1) after exposure to normal human epidermal keratinocytes (keratoCC) and normal human dermal fibroblasts (fibroCC) compared with reference melanoma cells used as control. These results suggest that downregulation of PS-1 expression may be an early event in melanoma cells localized in the skin, and this could facilitate Wnt signaling during the progression of melanoma to more aggressive disease.

To determine whether PS-1 was in fact decreased in aggressive melanoma, WB analysis for PS-1 expression was performed on lysates from the aggressive melanoma cell lines Sk-Mel28, A375, and C8161 (Supplementary Table S1). Western blot results showed that Sk-Mel28, A375, and C8161 expressed 52%, 29%, and 32%, respectively, of the PS-1 levels detected in cell lysates from the nonaggressive melanoma cell line WM1552C (Figure 1C). Since lower PS-1 expression could translate into increased Wnt activity, we also analyzed the lysates from the aggressive melanoma cells for P-β-catenin and found that Sk-Mel28, A375, and C8161 also expressed 54%, 71%, and 57%, respectively, of the P-β-catenin level detected in lysates from WM1552C (Figure 1D). These results suggest that the lower PS-1 levels detected in the aggressive melanoma cells than in the nonaggressive melanoma cells could explain the relatively higher Wnt signaling activity in the aggressive compared with the nonaggressive melanoma cells.

### 2.2. PS-1 Expression Was Significantly Lower in Advanced-Stage Than in Early-Stage Melanoma

**Figure 2:**
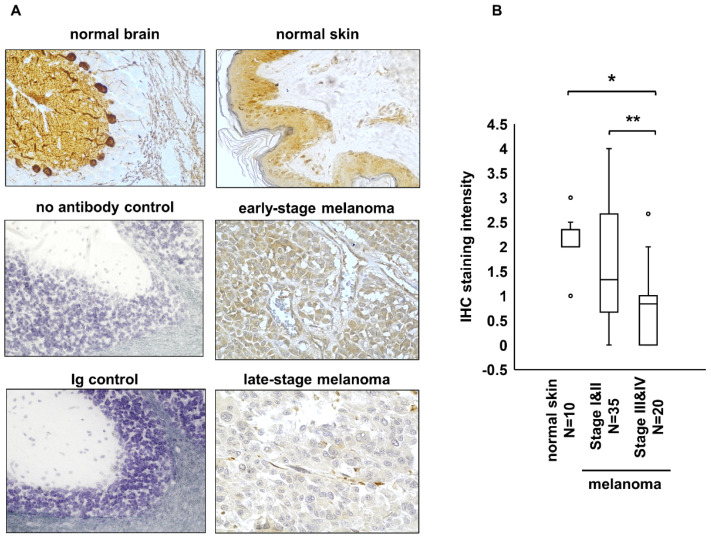
Immunohistochemistry for PS-1 in human melanoma tissue samples. (**A**) Representative IHC results showed poor staining for PS-1 in a late-stage melanoma compared with early-stage and normal skin tissue samples. Normal human skin and normal mouse brain tissue sections were used as positive control for PS-1 expression. IHC staining results obtained by omitting the primary antibody (no antibody control) or using an irrelevant isotype immunoglobulin (Ig control) represented negative controls. (**B**) Box plots show distribution of the IHC staining intensities for the cases of normal skin, early-stage melanoma (Stage I and II), and late-stage melanoma (Stage III and IV). These results showed that staining intensity for PS-1 was significantly lower in late-stage melanoma than in normal skin (* *p* < 0.01) and early-stage melanoma (** *p* < 0.02).

Our in vitro data showing decreased PS-1 expression in melanoma cells as they acquired more aggressive traits suggested that low PS-1 levels could represent a marker for melanoma aggressiveness in vivo. To investigate whether PS-1 expression in melanoma could have clinical significance, IHC staining was performed to assess PS-1 expression in tissue samples from human melanoma at various clinical stages and in normal skin (Supplementary Table S2), used as reference because it is known to ubiquitously express PS-1 [34] (Figure 2A). The mean IHC staining intensity results were grouped into normal skin, early-stage melanoma (Stages I and II), and advanced-stage melanoma (Stages III and IV) (Figure 2B). These data showed that the mean staining intensity for PS-1 was significantly lower in advanced-stage melanoma (0.77 +/− 0.17, N = 20) than in normal skin (2.1 +/− 0.16, N = 10) (*p* < 0.01) or early-stage melanoma (1.52 +/− 0.21, N = 35) (*p* < 0.02). There was no significant difference in mean staining intensity for PS-1 between early-stage melanoma and normal skin. These results suggest that low PS-1 expression could represent a biomarker for increased aggressiveness in melanoma.

### 2.3. Inhibition of γ-Secretase in Aggressive Melanoma Cells Led to Increased PS-1 Expression and Decreased Wnt Activity

**Figure 3:**
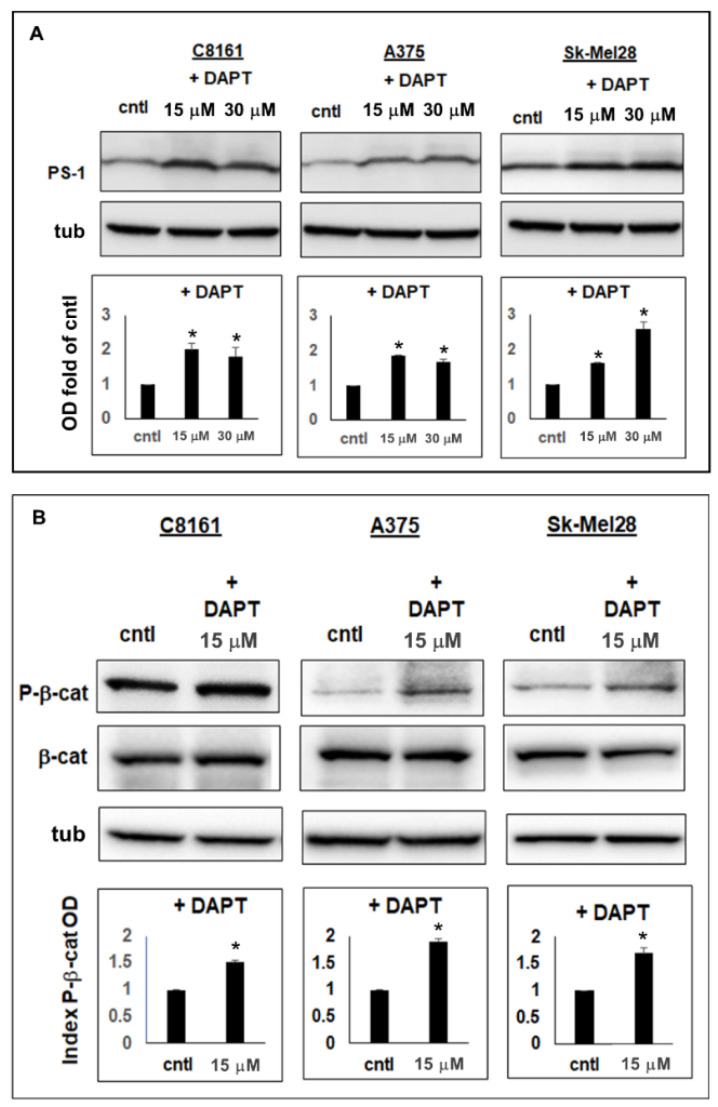
Effects of GSI treatment on PS-1 and P-β-catenin levels in aggressive melanoma cells. (**A**) Western blot results and relative densitometric analysis of bands showed that PS-1 expression increased in the aggressive human melanoma cell lines C8161, A375, and Sk-Mel28 when treated for 72 h with 15 μM or 30 μM GSI (DAPT) compared with vehicle-treated cells (cntl). (**B**) Western blot results and relative densitometric analysis of bands also showed that treatment of C8161, A375, and Sk-Mel28 for 72 h with 15 μM DAPT significantly increased levels of P-β-catenin (P-β-cat) compared with vehicle-treated cells (cntl) (* *p* < 0.05).

Gamma-secretase inhibition has been shown to increase PS-1 expression [13]. Therefore, to determine whether PS-1 expression could be increased in aggressive melanoma cells, we treated these cells with the GSI DAPT. Western blot analysis of lysates from C8161, A375, and Sk-Mel28 treated for 72 h with 15 μM or 30 μM DAPT showed a significant increase in PS-1 expression compared with vehicle-treated melanoma cells used as control (fold increase: C8161, 15 μM = 2.0 +/− 0.18, 30 μM = 1.8 +/− 0.26; A375, 15 μM = 1.9 +/− 0.02, 30 μM = 1.7 +/− 0.07; Sk-Mel28, 15 μM = 1.6 +/− 0.01, 30 μM = 2.6 +/− 0.2) (*p* < 0.05) (Figure 3A). Since PS-1 is known to downregulate Wnt/β-catenin signaling by increasing P-β-catenin levels [16,17,31], we analyzed lysates from the DAPT-treated cells for P-β-catenin expression. Results from WB analysis showed significant increases in P-β-catenin expression in C8161 (1.5 +/− 0.02-fold increase), A375 (1.9 +/− 0.05-fold increase), and Sk-Mel28 (1.7 +/− 0.1-fold increase) treated for 72 h with 15 μM DAPT, compared with vehicle-treated control (*p* < 0.05) (Figure 3B). These results demonstrated that increasing expression of PS-1 in aggressive melanoma cells with DAPT treatment could reduce Wnt signaling by enhancing P-β-catenin production.

### 2.4. Increased PS-1 Expression in DAPT-Treated Aggressive Melanoma Cells Was Associated with Reduced Cell Migration

**Figure 4:**
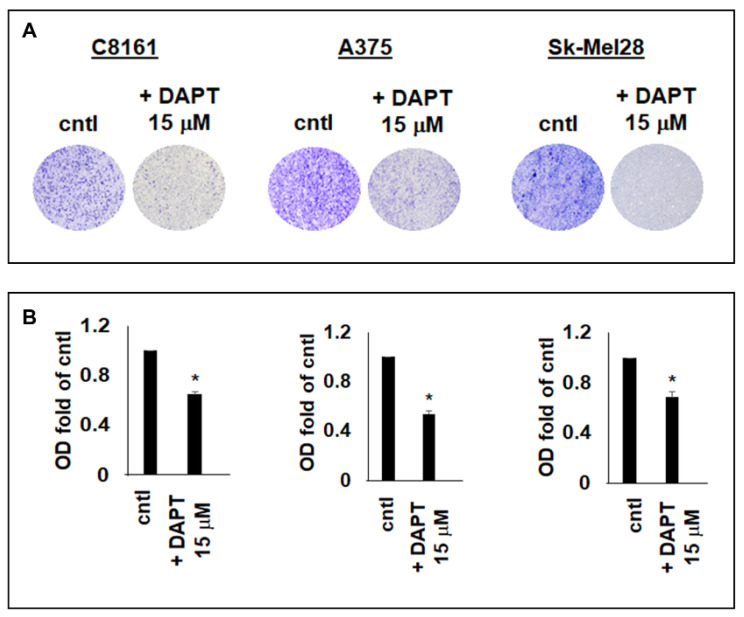
Cell migration assay in aggressive human melanoma cells treated with DAPT. (**A**) Microphotographic images from direct microscopic observation showed low staining intensity for reduced migration of C8161, A375, and Sk-Mel28 aggressive melanoma cells after 72 h treatment with 15 μM DAPT compared with vehicle-treated cells used as control (cntl). (**B**) Densitometric analysis of stain extracted from the migrated cells demonstrated the significant reduction in migration in the aggressive human melanoma cells lines after treatment for 72 h with 15 mM DAPT compared with control (* *p* < 0.05).

Wnt signaling has been shown to be involved in the migration and spread of aggressive melanoma [27]. Therefore, cell migration assays were performed to determine if the PS-1-associated decrease in Wnt activity in the DAPT-treated aggressive melanoma cells can lead to a functional effect. Our results showed that DAPT treatment of C8161, A375, and Sk-Mel28, in addition to the increased expressions of PS-1 and P-β-catenin shown above, also led to a significant reduction in cell migration compared with vehicle-treated control melanoma cells (C8161, −36% of control; A375, −46% of control; Sk-Mel28, −35% of control) (*p* < 0.05) (Figure 4A,B). Moreover, results from MTT cytotoxicity assay showed no significant difference in toxicity between the 15 μM DAPT treatment and control (Supplementary Figure S1). Thus, these findings suggest that the impaired migration of aggressive melanoma cells treated with the 15 μM DAPT was likely the result of a treatment-induced increase in PS-1, with a subsequent increase P-β-catenin levels leading to reduced Wnt signaling.

## 3. Discussion

Presenilins are multipass transmembrane proteins that include the highly homologous PS-1 and PS-2, which play a role in γ-secretase activity and other cell functions [11,12,13,15]. The main difference between PS-1 and PS-2 is that PS-1 is mostly expressed at the cell membrane level while PS-2 is mostly found within the cell and is associated with endosomes and lysosomes. Presenilins were first identified in screening for mutations in patients with familial Alzheimer [12,35]. In fact, it was found that aberrant PS-1 activity was associated with incomplete degradation of amyloid β-peptide, a known contributing factor to the onset of AD [12,13,36]. The interesting finding of lower incidence of certain cancers in patients with AD [9,10], in addition to the PS-1 regulatory function of Wnt signaling, has prompted studies to investigate the role of PS-1 in cancer biology. In some studies, PS-1 was shown to enhance carcinogenesis [37], while other studies, such as in nonmelanoma skin cancer, PS-1 functioned as a tumor suppressor [31]. Thus, the precise role of PS-1 in cancer remains unclear.

The main purpose of this study was to investigate the potential biologic role for PS-1 in melanoma. We recently showed that nonaggressive melanoma cells exposed to normal skin cells increased Wnt signaling and became more aggressive [33]. In this study, we also found that nonaggressive melanoma cells expressed lower PS-1 levels when exposed to normal skin cells compared with control. These results suggest that the increased Wnt signaling and aggressiveness we previously reported for these exposed nonaggressive melanoma cells could be the result of decreased PS-1 expression. In fact, screening cell lysates from several aggressive human melanoma cell lines revealed lower PS-1 expression and increased Wnt signaling, as demonstrated by the reduced P-β-catenin levels compared with nonaggressive melanoma cells. These findings were consistent with previous reports showing the association between loss of PS-1 and increased Wnt signaling and tumorigenesis [31]. To investigate whether PS-1 expression correlated with melanoma aggressiveness in vivo, we performed IHC on human melanoma tissue samples at different clinical stages and found that PS-1 expression was significantly lower in advanced-stage than in early-stage melanoma, demonstrating for the first time that PS-1 could be used as a marker of aggressive melanoma. Moreover, since AD has been associated with aberrant PS-1 function and/or expression [12], our finding that PS-1 levels were lower in patients with certain types of melanoma also provides a possible explanation as to why the incidence of AD is lower in patients with melanoma [9,10].

Given the role of Wnt during tumor progression, it remains an attractive target for anticancer therapy. Thus, preventing Wnt activity in early-stage melanoma could reduce the chances of metastatic spread. For this reason, we explored whether, by increasing PS-1 expression, we could reduce melanoma aggressiveness. DAPT is a GSI previously shown to increase PS-1 expression in treated cells [13]. We reported that aggressive melanoma cells treated for 72 h with 15 μM or 30 μM DAPT had increased PS-1 and P-β-catenin levels. Interestingly, although there was a trend towards lower total β-catenin levels in the treated melanoma cells, this was not significant (data not shown). This suggests that additional time may have been required for our treated aggressive melanoma cells to continue with ubiquitination and proteasomal degradation of the P-β-catenin, which would have resulted in further reduction in total β-catenin. Nevertheless, since only active, nonphosphorylated/nonubiquitinated β-catenin can translocate to the nucleus to induce Wnt-dependent gene transcription [38], the increased P-β-catenin Ser33/37/Thr41 expression observed in our DAPT-treated aggressive melanoma cells could, in itself, be sufficient to negatively affect Wnt-associated functions. In fact, treatment of aggressive melanoma cells with 15 μM DAPT significantly reduced cell migration, a well-known Wnt-regulated activity [16,26], compared with control.

Studies investigating GSI to treat cancers, including melanoma have shown conflicting results. For example, while GSI treatment led to decreased self-renewal and stemness of melanoma cells, long-term treatment facilitated tumor growth, especially in patients with advanced metastatic disease [39]. One explanation as to the inefficacy of certain GSIs in cancer is the negative effects that GSIs have on host antitumor immune activity [40,41]. Another possible explanation of the conflicting data on GSI effects in cancer is the fact that different GSIs have varying pharmacologic and functional profiles depending on the concentrations used [42]. In fact, most clinical trials involve administering relatively high doses of GSI to treat metastatic disease. Some of these doses reach plasma levels that are several orders of magnitude greater than the concentrations used in our study to increase PS-1 expression [43,44]. Thus, treatment of early-stage melanoma with GSIs for enhancing PS-1 expression may require lower doses or shorter treatment regimens, thereby reducing the chances of undesired effects that are associated with longer-term or higher-dose treatment regimes. Our results showed that treatment of aggressive melanoma cells with just 15 μM DAPT could increase PS-1 levels and decrease Wnt signaling and cell migration, suggesting that metastatic potential in melanoma could be reduced by increasing PS-1 expression. Thus, future research aimed at developing treatment strategies to enhance PS-1 expression in aggressive melanoma may reduce melanoma progression.

In summary, our results demonstrated that decreased PS-1 expression was associated with melanoma aggressiveness both in vitro and in human samples. We showed that in aggressive melanoma cells, Wnt signaling activity may increase because of low PS-1 expression. Moreover, we demonstrated that by enhancing PS-1 expression in aggressive melanoma cells, Wnt signaling activity was reduced, and a less migratory and more static phenotype was acquired. Ultimately, these results confirm the importance PS-1in regulating Wnt signaling and suggest a novel role for PS-1 as a biomarker for aggressiveness with therapeutic potential in melanoma.

## 4. Materials and Methods

### 4.1. Cell Cultures

The following cell lines were used (Supplementary Table S1): poorly aggressive melanoma cell lines WM1552C (Rockland Immunochemicals, Limerick, PA, USA) and UACC1273 (a generous gift from Dr. Richard Seftor, University of West Virginia, WV, USA) and aggressive melanoma cell lines C8161 (Dr. Richard Seftor, University of West Virginia), A375 (CRL-1619, ATCC, Manassas, VA, USA), and Sk-Mel28 (HTB-72, ATCC, USA). All cells were maintained in RPMI1640 media (GenClone, San Diego, CA, USA) except for Sk-Mel28 (EMEM, ATCC) and supplemented with 5% FBS (Seradigm, Batavia, IL, USA). Epidermal melanocytes (PCS-200-013, ATCC), epidermal keratinocytes (PCS-200-010, ATCC), and dermal fibroblasts (PCS-201-012, ATCC, USA) were grown according to ATCC specifications. All cell lines were incubated at 37 °C and 5% CO_2_.

### 4.2. Serial Coculture

Nonaggressive melanoma cells were exposed in sequence first to normal human epidermal keratinocytes and then to normal dermal fibroblasts as previously described [33]. Briefly, WM1552C or UACC1273 were seeded into transwells (Corning, Corning, NY, USA) containing inserts with porous membranes that allowed for molecular crosstalk between melanoma and normal cells without the cells coming into physical contact. These inserts were then placed in 6-well plates containing the normal epidermal keratinocytes or dermal fibroblasts. The nonaggressive melanoma cells were allowed to grow in sequence first for 24 h with the normal keratinocytes and then for 24 h with the normal dermal fibroblasts. After this coculture sequence, the melanoma cells were harvested for Western blot (WB) protein analysis.

### 4.3. Western Blotting

WB analysis was performed as previously described [45]. Briefly, lysates from melanoma cells exposed to normal skin cells or from treated melanoma cells were obtained using standard RIPA buffer (Pierce, Waltham, MA, USA) containing protease and phosphatase inhibitors (Pierce). SDS–PAGE electrophoresis was used to separate 30 µg of protein per sample, which was then transferred to PVDF membranes (Millipore, Burlington, MA, USA). Afterwards, membranes were washed 3 times with TBST (tris-buffered saline with 0.1% Tween 20) and then blocked with 5% nonfat dry milk or 5% bovine serum albumin for 1 h at room temperature (RT). Membranes were then incubated with adequate dilutions of primary antibody in blocking buffer overnight at 4 °C. The listed antibodies/dilutions were used: goat anti-presenilin/1:400 (AF166, R&D Systems, Minneapolis, MN, USA); rabbit anti-P-β-catenin (Ser33/37/Thr41)/1:1000 (9561, Cell Signaling, Danvers, MA, USA); mouse anti-β-catenin/1:1000 (NBP1-54467, Novus Biologicals, Centennial, CO, USA); and rabbit anti-α-tubulin/1:5000 (2144, Cell Signaling). After incubation, membranes were washed and then incubated for 1 h with adequate dilutions of conjugated secondary antibodies: antimouse 1:5000 (NA931, GE Amersham, Marlborough, MA, USA); antirabbit 1:5000 (NA934, GE Amersham); and antigoat 1:5000 (HADF109, R&D Systems). After washing, membranes were incubated in ECL (GE Amersham) for 5 min and images captured using Bio-Rad Universal Chemidoc system.

### 4.4. Drug Treatment

In a recent study reporting the effects of g-secretase inhibition in melanoma cells, the authors showed that treatment of cells with DAPT had negligible toxic effects at concentrations ranging from 5 to 60 μM [39]. Based on these results, for this study, it was decided to treat our aggressive melanoma cells with 15 μM and 30 μM DAPT to determine whether these concentrations could have effects on PS-1 expression. Thus, C8161, Sk-Mel28, and UACC1273 were treated were washed once in PBS and then treated with a final concentration of 15 μM or 30 μM DAPT (A8200, Apexbio, Houston, TX, USA) or vehicle control for 72 h. A standard 4,5-dimethylthiazol-2-yl-2,5-diphenyltetrazolium bromide (MTT) cell viability assay was performed as previously described [45] to determine whether the concentrations of DAPT used resulted in cellular toxicity. Lysates were also obtained from the treated cells to perform WB for the detection of PS-1 and P-β-catenin, as described above.

### 4.5. Immunohistochemistry

Immunohistochemistry (IHC) staining was performed as previously described [45] to assess PS-1 expression in commercially available paraffin-embedded tissue microarray containing normal human skin and melanoma tissue samples at different clinical stages (Me1002b, USBiomax, Rockville, MD, USA). Briefly, after hydration, antigen retrieval, and blocking of endogenous peroxidases and nonspecific binding sites, slides were incubated with primary rabbit anti-PS-1 antibody (1:100, LS-C800364-100, LSBio, Seattle, WA, USA) for 1 h. Then, slides were washed and incubated with secondary antirabbit antibody (PK-4001, Vector Laboratories, Burlingame, CA, USA) for 30 min, washed and incubated with avidin–biotin complex reagent (PK-4001, Vector Laboratories, USA) for 30 min, and washed again and treated with DAB substrate (SK-4105, Vector Laboratories, Burlingame, CA, USA) for stain development. Slides were then washed with distilled water, counterstained with hematoxylin, washed, and dehydrated in a graded ethanol series followed by Histo-Clear. Slides were finally mounted with Aqua-Poly mounting media (Polysciences, Warrington, PA, USA) and observed under light microscopy. To determine appropriate sample size for our IHC study, we assumed 100,000 melanoma patients in the United States [46] and a confidence level of 95% for a difference in PS-1 expression of at least 1/3 (33%). These assumptions indicated that at least 9 samples per group should be analyzed. Since the PS-1 antibody used was specific for mouse and human PS-1, tissue sections from archival paraffin-embedded mouse brain (generous gift from Dr. Maria Traka, Midwestern University, Downers Grove, IL, USA) were used as positive control for IHC PS-1 expression.

### 4.6. Migration Assay

Migration assay was performed as previously described [45]. Briefly, cells were seeded into 3.0 μm pore, 24-well transwell inserts (Falcon, Corning, NY, USA) at 150,000 cells/transwell insert and incubated overnight at 37 °C and 5% CO_2_. Transwell inserts were then cleansed of nonmigrated residue with moist cotton swabs and then further washed in PBS. Transwell inserts were then transferred to wells with crystal violet solution and incubated for 10 min at RT. To extract the stain, stained transwells were then washed with PBS and incubated in wells with 10% acetic acid solution for 10 min. Finally, stain from each sample was transferred to a 96-well plate for OD reading at 590 nm. C8161, A375, and Sk-Mel28 cells were treated with DAPT or vehicle for 72 h prior to the actual migration assay. To acquire images of cell migration, transwell inserts were washed as stated above, and the cells were fixed in methanol for 20 min before staining with crystal violet for 30 s. Next, transwell inserts were washed with PBS. This was followed by cutting of the growth surface (bottom), which was finally mounted on a slide with Cytoseal-60 (Thermo Scientific, Waltham, MA, USA) and observed under light microscopy. A standard 4,5-dimethylthiazol-2-yl-2,5-diphenyltetrazolium bromide (MTT) assay was performed as previously described [45] to determine whether the concentration of DAPT used for the migration assay could result in confounding cytotoxicity.

### 4.7. Statistical Analysis

GraphPad statistical software was used to perform *t*-tests to compare the mean values (+/− standard error of the mean (SEM)) calculated from a minimum of triplicate results from at least two independent experiments between treated and untreated cells or between cocultured and reference control melanoma cells. GraphPad statistical software was also used to perform *t*-tests to compare the mean intensity IHC staining results (+/−standard deviation of the mean (SD)) among normal skin and early- and advanced-stage melanoma samples. Results showing *p* values of less than 0.05 (*p* < 0.05) was considered statistically significant.

## Data Availability

Data is contained within the article or supplementary material. The data presented in this study are available on request from the corresponding author.

## Supplementary Materials

The following supporting information can be downloaded at: https://www.mdpi.com/article/10.3390/ijms23094904/s1.
