# Supplementary material for: Role of Presenilin-1 in Aggressive Human Melanoma
Source: Int J Mol Sci. 2022 Apr 28;23(9):4904. doi: 10.3390/ijms23094904 (PMC9099829; doi:10.3390/ijms23094904)
Supplement: Supplementary file 1 [file ijms-23-04904-s001.zip › Supplementary Table S2.pdf]

Case	Diagnosis	TNM	Stage	PS-1IHC score
1	normal skin	-	-	2
2	normal skin	-	-	2
3	normal skin	-	-	2
4	normal skin	-	-	2.5
5	normal skin	-	-	3
6	normal skin	-	-	2.3
7	normal skin	-	-	1
8	normal skin	-	-	2.3
9	normal skin	-	-	2
10	normal skin	-	-	2
11	melanoma	T1N0M0	1A	0.67
12	melanoma	T2aN0M0	1B	0
13	melanoma	T2aN0M0	1B	1.33
14	melanoma	T2aN0M0	1B	2.67
15	melanoma	T2aN0M0	1B	3.67
16	melanoma	T3N0M0	2A	0
17	melanoma	T4N0M0	2B	1.17
18	melanoma	T4N0M0	2B	1.17
19	melanoma	T4N0M0	2B	0.33
20	melanoma	T4N0M0	2B	0.17
21	melanoma	T4N0M0	2B	0.17
22	melanoma	T4N0M0	2B	2.67
23	melanoma	T4N0M0	2B	1
24	melanoma	T4N0M0	2B	3.33
25	melanoma	T4N0M0	2B	2.33
26	melanoma	T4N0M0	2B	2.33
27	melanoma	T4N0M0	2B	3.67
28	melanoma	T4N0M0	2B	0
29	melanoma	T4N0M0	2B	1.67
30	melanoma	T4N0M0	2B	4
31	melanoma	T4N0M0	2B	0
32	melanoma	T4N0M0	2B	1.33
33	melanoma	T4N0M0	2B	0.67
34	melanoma	T4N0M0	2B	1
35	melanoma	T4N0M0	2B	0
36	melanoma	T4N0M0	2B	0
37	melanoma	T4N0M0	2B	1.67
38	melanoma	T4N0M0	2B	3
39	melanoma	T4N0M0	2B	2.33
40	melanoma	T4N0M0	2B	3
41	melanoma	T4N0M0	2B	1
42	melanoma	T4N0M0	2B	1.33
43	melanoma	T4N0M0	2B	3
44	melanoma	T4N0M0	2B	0.67
45	melanoma	T4N0M0	2B	2
46	melanoma	T4N1M0	3	0
47	melanoma	T4N1M0	3	0.33
48	melanoma	T4N2M0	3	1
49	melanoma	T4N2M0	3	1
50	melanoma	T4N1M0	3	0.67
51	melanoma	T4N1M0	3	0.67
52	melanoma	T4N1M0	3	2
53	melanoma	T4N1M0	3	2
54	melanoma	T4N2M0	3	2.67
55	melanoma	T4N0M1	4	1
56	melanoma	T4N0M1	4	1
57	melanoma	na	4	0
58	melanoma	na	4	0
59	melanoma	na	4	0
60	melanoma	na	4	0
61	melanoma	na	4	0
62	melanoma	na	4	0
63	melanoma	na	4	1
64	melanoma	na	4	1
65	melanoma	na	4	1
