# Supplementary figures and images for: Role of Presenilin-1 in Aggressive Human Melanoma
Source: Int J Mol Sci. 2022 Apr 28;23(9):4904. doi: 10.3390/ijms23094904 (PMC9099829; doi:10.3390/ijms23094904)

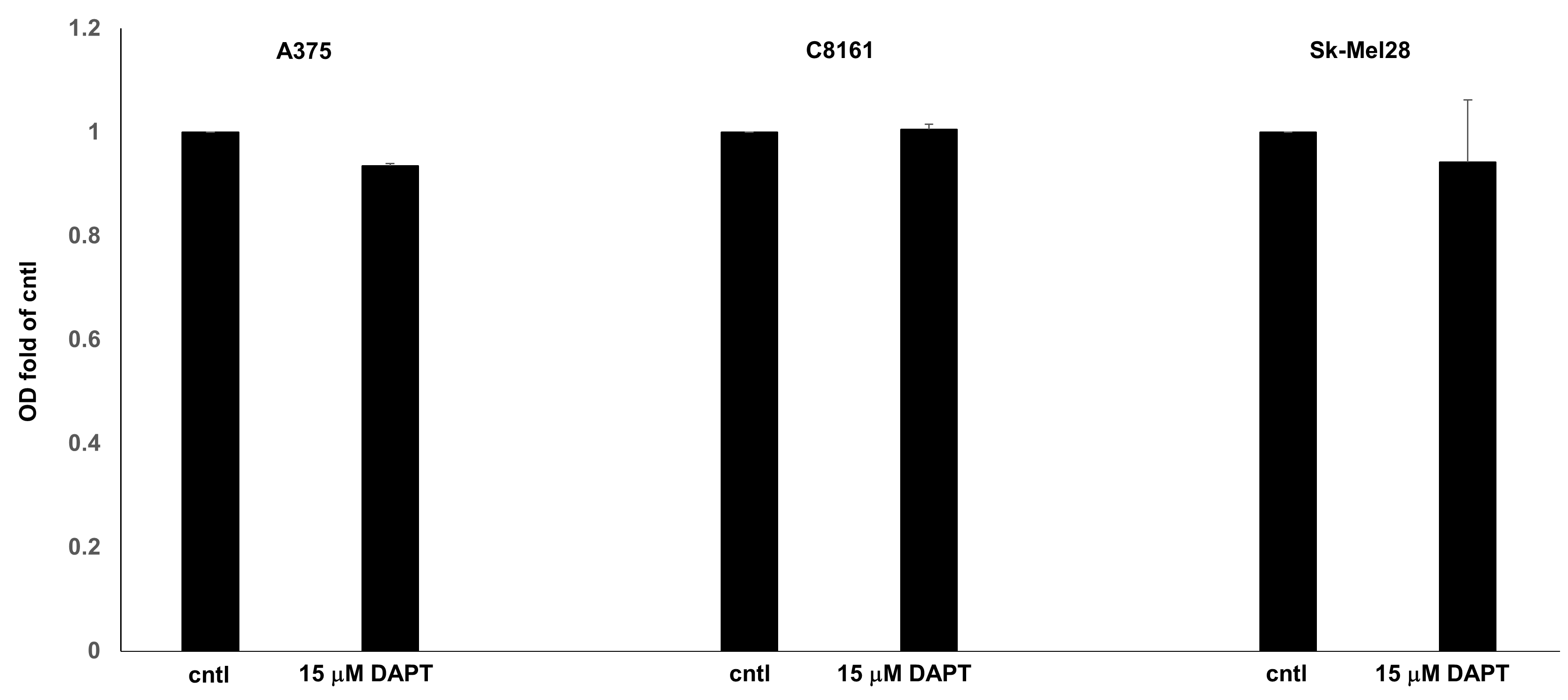

Supplement: Supplementary file 1 [file ijms-23-04904-s001.zip › Supplementary Figure S1.tif]

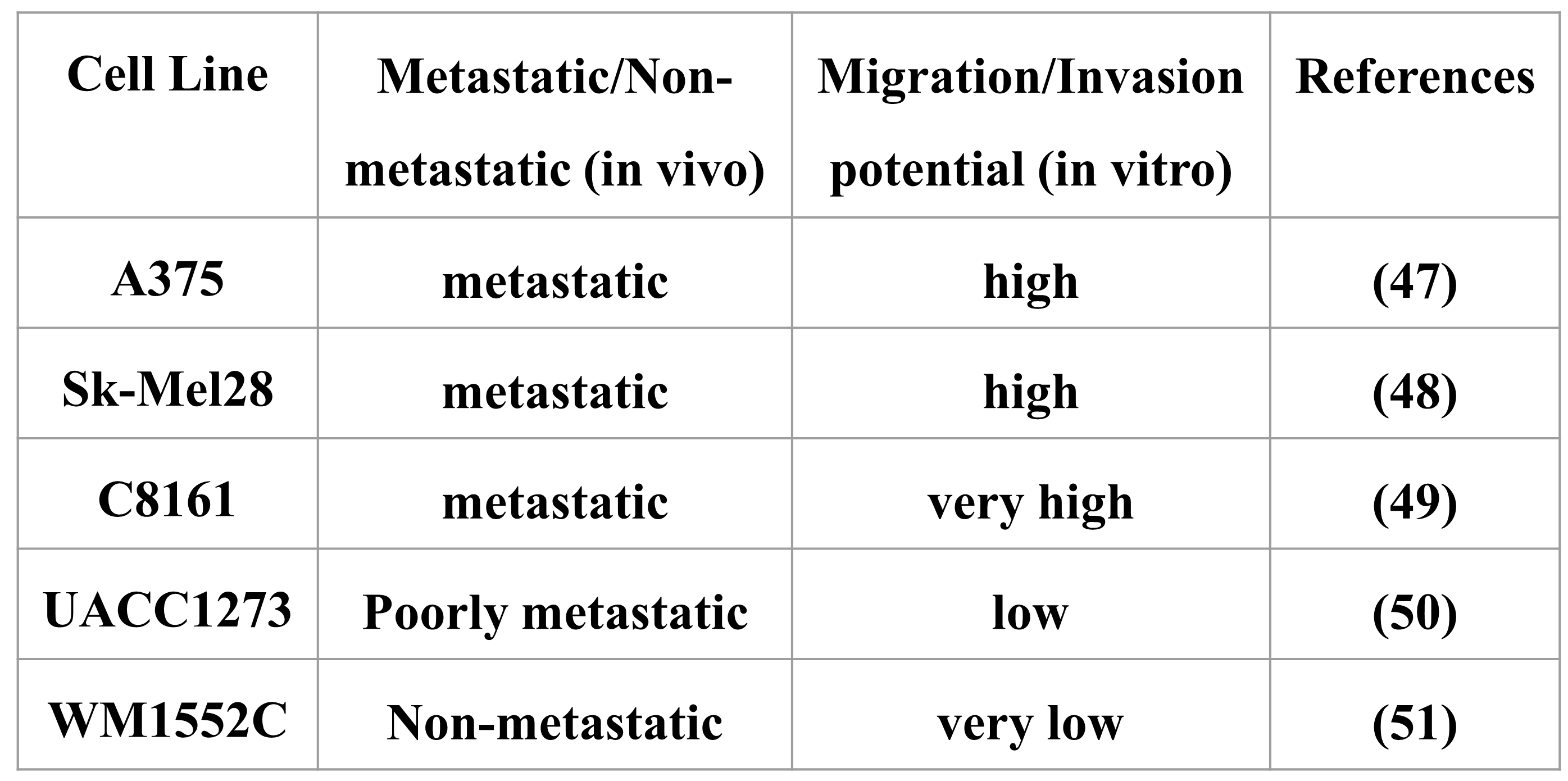

Supplement: Supplementary file 1 [file ijms-23-04904-s001.zip › Supplementary Table S1.tif]
